# Effect of alternating day and night temperature on short day-induced bud set and subsequent bud burst in long days in Norway spruce

**DOI:** 10.3389/fpls.2014.00691

**Published:** 2014-12-09

**Authors:** Jorunn E. Olsen, YeonKyeong Lee, Olavi Junttila

**Affiliations:** ^1^Department of Plant Sciences, Norwegian University of Life SciencesÅs, Norway; ^2^Department of Arctic and Marine Biology, University of TromsøTromsø, Norway

**Keywords:** bud burst, bud set, day temperature, dormancy, long day, night temperature, Norway spruce, short day

## Abstract

Young seedlings of the conifer Norway spruce exhibit short day (SD)-induced cessation of apical growth and bud set. Although different, constant temperatures under SD are known to modulate timing of bud set and depth of dormancy with development of deeper dormancy under higher compared to lower temperature, systematic studies of effects of alternating day (DT) and night temperatures (NT) are limited. To shed light on this, seedlings of different provenances of Norway spruce were exposed to a wide range of DT-NT combinations during bud development, followed by transfer to forcing conditions of long days (LD) and 18°C, directly or after different periods of chilling. Although no specific effect of alternating DT/NT was found, the results demonstrate that the effects of DT under SD on bud set and subsequent bud break are significantly modified by NT in a complex way. The effects on bud break persisted after chilling. Since time to bud set correlated with the daily mean temperature under SD at DTs of 18 and 21°C, but not a DT of 15°C, time to bud set apparently also depend on the specific DT, implying that the effect of NT depends on the actual DT. Although higher temperature under SD generally results in later bud break after transfer to forcing conditions, the fastest bud flush was observed at intermediate NTs. This might be due to a bud break-hastening chilling effect of intermediate compared to higher temperatures, and delayed bud development to a stage where bud burst can occur, under lower temperatures. Also, time to bud burst in un-chilled seedlings decreased with increasing SD-duration, suggesting that bud development must reach a certain stage before the processes leading to bud burst are initiated. The present results also indicate that low temperature during bud development had a larger effect on the most southern compared to the most northern provenance studied. Decreasing time to bud burst was observed with increasing northern latitude of origin in un-chilled as well as chilled plants. In conclusion, being a highly temperature-dependent process, bud development is strongly delayed by low temperature, and the effects of DT is significantly modified by NT in a complex manner.

## INTRODUCTION

To survive the winter, temperate and boreal zone trees must cease their growth, develop dormant winter buds and acquire cold hardiness in time before the onset of the harsh conditions. In young individuals exhibiting a free (indeterminate) growth pattern, this is well known to occur in response to a photoperiod shorter than a critical length and change in light quality, which is sensed by the phytochrome system (Wareing, 1956; Nitsch, 1957; Olsen et al., 1997a; Clapham et al., 1998; Mølmann et al., 2006). Furthermore, to avoid freezing during unstable temperature conditions in the spring, the timing of bud flush and de-hardening is of outmost importance. In addition to the significance of the light climate, particularly in dormancy induction, temperature is well known to play important roles in several aspects of growth-dormancy transitions and development of cold hardiness, and has been shown to modify the sensitivity to photoperiod and influence the duration of bud formation (Olsen, 2010; Tanino et al., 2010, 2014; Rohde et al., 2011; Cooke et al., 2012; Junttila and Hänninen, 2012; and references therein).

Furthermore, in several deciduous species [e.g., white birch (*Betula pendula*), downy birch (*B. pubescens*), black alder (*Alnus glutinosa*) Norway maple (*Acer platanoides*) and *Populus*] as well as conifer species like Norway spruce (*Picea abies*), high temperature under exposure to short days (SD) has been reported to result in accelerated winter bud formation and dormancy acquisition as well as deeper dormancy compared to relatively low temperatures (Westergaard and Eriksen, 1997; Heide, 2003; Junttila et al., 2003; Søgaard et al., 2008; Kalcsits et al., 2009a). Some woody species of the rose family, such as apple (*Malus domestica*) and pear (*Pyrus communis*) are insensitive to photoperiod, and instead show growth cessation and formation of dormant winter buds in response to relatively low temperature (lower than 12°C; Heide and Prestrud, 2005). In still other species, like *Prunus*, sensitivity to photoperiod depends on temperature (Heide, 2008). At relatively high, constant temperatures such as 21°C, such plants were found to be insensitive to photoperiod, whereas at lower temperatures the response to photoperiod varied with species and cultivar. Furthermore, northern ecotypes of woody species such as Norway spruce, downy birch, hybrid aspen (*Populus tremula x tremuloides*) and other hybrid poplars, bay willow (*Salix pentandra*) and dogwood (*Cornus sericea*) were shown to induce bud set in response to low temperature even under long day (LD) conditions (Håbjørg, 1972a,b; Heide, 1974; Junttila, 1980; Mølmann et al., 2005; Svendsen et al., 2007). In this respect, generally a low night temperature (NT) appears very efficient. Thus, for initiation of dormancy in young seedlings of tree species with a free growth pattern, two different response pathways seem to exist, one induced by warm temperature and short photoperiod, and another induced by low-temperature stress in northern and high altitude areas. This apparently ensures flexibility in the ability to maximise growth and reduce the risk of freezing injury (Tanino et al., 2010).

Chilling is commonly needed to alleviate dormancy in a wide range of woody species. The duration of chilling required, varies considerably between species and even populations within a species, with chilling requirement often decreasing with increasing latitude (McCreary et al., 1990; Myking and Heide, 1995; Hannerz et al., 2003; Cooke et al., 2012; Junttila and Hänninen, 2012). Chilling requirement is usually defined as the minimum chilling making buds able to burst after return to warm, growth-permissive conditions. However, in Norway spruce, and at least some other conifers, chilling hastens bud break but is not required in young seedlings (Nienstaedt, 1967; Worrall and Mergen, 1967; Søgaard et al., 2008), indicating a quite shallow dormancy at least at this stage of such species. Irrespective of whether chilling is required or not, the quiescent bud will flush after exposure to a cumulative heat sum. The required heat sum varies between and within species, and is also affected by the state of dormancy and the length of the chilling period, with generally higher heat sum requirement after shorter periods of chilling (Junttila and Hänninen, 2012).

The current scenario of global warming due to climate change is characterised not only by increasing mean temperatures, but also by decreasing differences between day (DT) and NT due to increasing NT, particularly in northern areas (Karl et al., 1993; Easterling et al., 1997; Beaubien and Hamann, 2011). It has been suggested that NT might affect growth cessation, bud set and dormancy more than DT (Tanino et al., 2010 and references therein). In addition to the aforementioned effect of low NT in inducing growth cessation and buds in northern ecotypes, warm nights have been shown to accelerate growth cessation and dormancy induction in hybrid poplars of more southern origin (Kalcsits et al., 2009a). However, most studies on bud set and bud break under controlled conditions have applied constant temperatures, and few investigations have systematically addressed effects of diurnal temperature fluctuation. Existing studies have generally included few DT-NT combinations only (see e.g., Tanino et al., 2010 for review).

In previous studies of the conifer Norway spruce, higher constant temperatures during SD exposure resulted in accelerated bud set and deeper dormancy compared to lower constant temperatures (Søgaard et al., 2008). To shed light also on the effect of diurnal temperature variation in Norway spruce, using a wide range of DT-NT combinations we aimed at investigating the effects of alternating DT and NT on winter bud formation and depth of dormancy, measured as time to bud burst. Thus, seedlings of different provenances were exposed to alternating DT and NT during SD-induced bud development, followed by transfer after different SD durations, to forcing conditions of LD and 18°C, either directly or after different periods of chilling. Furthermore, to assess the effect of different temperatures under SD on the anatomy of terminal buds, microscopy studies were also conducted.

## MATERIALS AND METHODS

### PLANT MATERIALS, GROWING CONDITIONS AND EXPERIMENTAL TREATMENTS

Experiments with three provenances of Norway spruce [*Picea abies* L. (Karst)] seedlings were conducted in the phytothrone at the University of Tromsø, Norway (69°39′N, 18°55′E). Temperature was controlled to ± 0.5°C, and relative air humidity adjusted to 0.5 kPa water vapour pressure deficit. Seeds were sown in late May (2006) in 12 cm pots (volume 0.8 l) in a fertilized peat: perlite (7:3) (v/v) mixture. The following three provenances from the elevation 0–149 m a s l in Norway were used in the experiments: provenance P1, orginating from Rana (66°25′N, 14°30′E), M1, originating from Namskogan (64°50′N, 13°00′E) and F1, originating from Arendal/Tvedstrand (58°35′N, 8°50′E). After germination the seedlings were thinned to 6–7 uniform seedlings per pot. For each bud break treatment and provenance two pots were used, whereas for bud set recording 12 pots were used for each provenance. Seedlings were raised at 18°C in natural light in the phytothrone (22–24 h photoperiod) until the experimental treatments started. Seedlings were watered daily and fertilized weekly with a complete nutrient solution. Great care was taken to avoid differences in water and nutrient status between pots.

From August 1, the seedlings were exposed to SDs of 12 h photoperiod in natural daylight from 08.00–20.00 in a phytothrone room. Curtains were used to close out natural light when photoperiod exceeded 12 h. The natural light was supplemented by artificial light (Phillips TLD 480 daylight fluorescent tubes, Eindhoven, the Netherlands) when natural solar irradiance was less than 130–140 μmol m^-2^ s^-1^ at plant height. During the SD treatment, seedlings were exposed to different combinations of day (15, 18, or 21°C; DT) and night temperature (NT; 6, 9, 12, 15, 18, 21°C). The DT of 15°C was combined with NT of 6, 9, 12, and 15°C, the 18°C DT with NT of 6, 9, 12, 15, and 18°C, and the DT of 21°C with NT of 6, 9, 12, 15, 18, and 21°C. Thus, altogether 15 different DT-NT combinations were given under the SD exposure. These different DT-NT combinations gave the daily mean temperatures (DMT) 10.5°C (DT/NT 15/6°C), 12°C (DT/NT 15/9°C, 18/6°C), 13.5°C (DT/NT 15/12°C, 18/9°C, 21/6°C), 15°C (DT/NT 15/15°C, 18/12°C, 21/9°C), 16.5°C (DT/NT 18/15°C, 21/12°C), 18°C (DT/NT 18/18°C, 21/15°C), 19.5 (DT/NT 21/18°C), 21 (DT/NT 21/21°C; **Table 1**). For different subsets of plants, these 15 different DT-NT combinations given under SD were provided for 4, 8, or 12 weeks. Thereafter, seedlings were transferred to forcing conditions directly (plants exposed to 4, 8, or 12 weeks SD) or after chilling periods of 3, 6, or 9 weeks at 4°C in darkness (plants exposed to 12 weeks of SD). Forcing with observation of bud burst was performed under controlled environmental conditions in growth chambers at a temperature of 18°C, and continuous light at about 130–140 μmol m^-2^ s^-1^ from fluorescent lamps (Phillips TLD 480) and incandescent lamps (10 μmol m^-2^ s^-1^; Osram; Munich, Germany). Altogether about 3400 plants were included in the study. For recording of bud set totally 72–84 plants (12 pots) were used for each DT-NT combination for each of the three provenances. To investigate the effect of SD duration (4, 8 or, 12 weeks) and duration of chilling (0, 3, 6, 9 weeks) on subsequent bud break after re-transfer to forcing conditions, 12–14 plants (2 pots) per provenance were used in each case (for each DT-NT combination).

**Table 1:** The 15 different day (DT) and night temperature (NT) combinations provided to Norway spruce seedlings under short day (SD) treatment and the daily mean temperature for each combination.

Day temperature (°)	Night temperature (°)	Daily mean temperature (°)
15	6	10.5
15	9	12.0
15	12	13.5
15	15	15.0
18	6	12.0
18	9	13.5
18	12	15.0
18	15	16.5
18	18	18.0
21	6	13.5
21	9	15.0
21	12	16.5
21	15	18.0
21	18	19.5
21	21	21.0

*The treatments were given for 4, 8, or 12 weeks before transfer to forcing conditions of long days and constant 18°C, either directly (after 4, 8, or 12 weeks of SD and DT-NT treatments) or after chilling at 4°C for 3, 6, or 9 weeks (after 12 weeks of SD and DT-NT treatments).*

### ANALYSIS OF TERMINAL BUDS BY MICROSCOPY

To assess the effect of temperature during SD on bud anatomy, particularly with respect to number of leaf initials, terminal buds were harvested after 8 weeks of SD treatment of plants exposed to 12°C/6°C DT/NT or constant temperatures of 9°C, 12°C, or 18°C (all other conditions as described above) and fixed in FAA (37–40% formaldehyde/acetic acid/50% ethanol, 2:1:17, by vol.). Also, a subset of plants were after the 8 weeks of SD at these temperatures transferred to LD of 24 h photoperiod (forcing conditions as described above), and terminal buds were harvested after one and 2 weeks under LD. After fixation, the shoot tips were washed with phosphate buffered saline solution (PBS, pH 7.0), followed by dehydration through an aqueous ethanol series and embedding in LR White resin (London Resin, Basingstoke, UK) at 50°C overnight, all according to Lee et al. (2008). The embedded materials were then sectioned longitudinally in 1 μm thick sections and stained with toluidine blue (Sigma-Aldrich, St. Louis, MO, USA). After rinsing and mounting (Lee et al., 2008), the embedded, stained sections were examined for number of leaf initials in the terminal buds using a light microscope (Leica, Wetzlar, Germany). For each temperature and daylength treatment (8 weeks SD, 1 and 2 weeks of LD after 8 weeks SD), three plants were used from each of the three provenances studied.

### STATISTICAL ANALYSES

Effect of the temperature treatments on time to bud set and bud burst were tested statistically using analysis of variance (ANOVA), followed by Fishers PLSD test (*p*≤ 0.05; Statview 4.0 Abascus Concepts, Inc., Berkley, CA, USA). Factorial analyses were done with respect to effects of DT and NT on bud set as well as effects of DT and NT, duration of SD treatment and chilling on bud break. The analyses were based on days to 50% visible bud and days to 50% bud burst (according to Søgaard et al., 2008). Since there was generally larger variation within pots than between pots, plant was used as the experimental unit. As mentioned above, great care was taken to avoid significant differences in water and nutrient status between pots, and due to their small size, seedlings grown in the same pot did not shadow each other and the likelihood of significant root competition between plants was very small. Effect of temperature treatment during SD (after 8 weeks of SD as well as one and 2 weeks after transfer to LD following 8 weeks of SD) on number of leaf initials within terminal buds of the three studied provenances was analysed using ANOVA (General linear model procedure) and Tukey’s multiple comparison test (*p* ≤ 0.05) using Mintab Version 16 (Minitab Inc., State College, PA, USA).

## RESULTS

### EFFECT OF DAY–NIGHT TEMPERATURE COMBINATIONS DURING SD ON BUD SET

The effect of different combinations of DT (15, 18, and 21°C) and NT (6, 9, 12, 15, 18, and 21°C; NT up to that of the DT) on SD-induced terminal bud formation and subsequent bud burst, were investigated in provenances originating from 58, 64, and 66°N in Norway.

**FIGURE 1:**
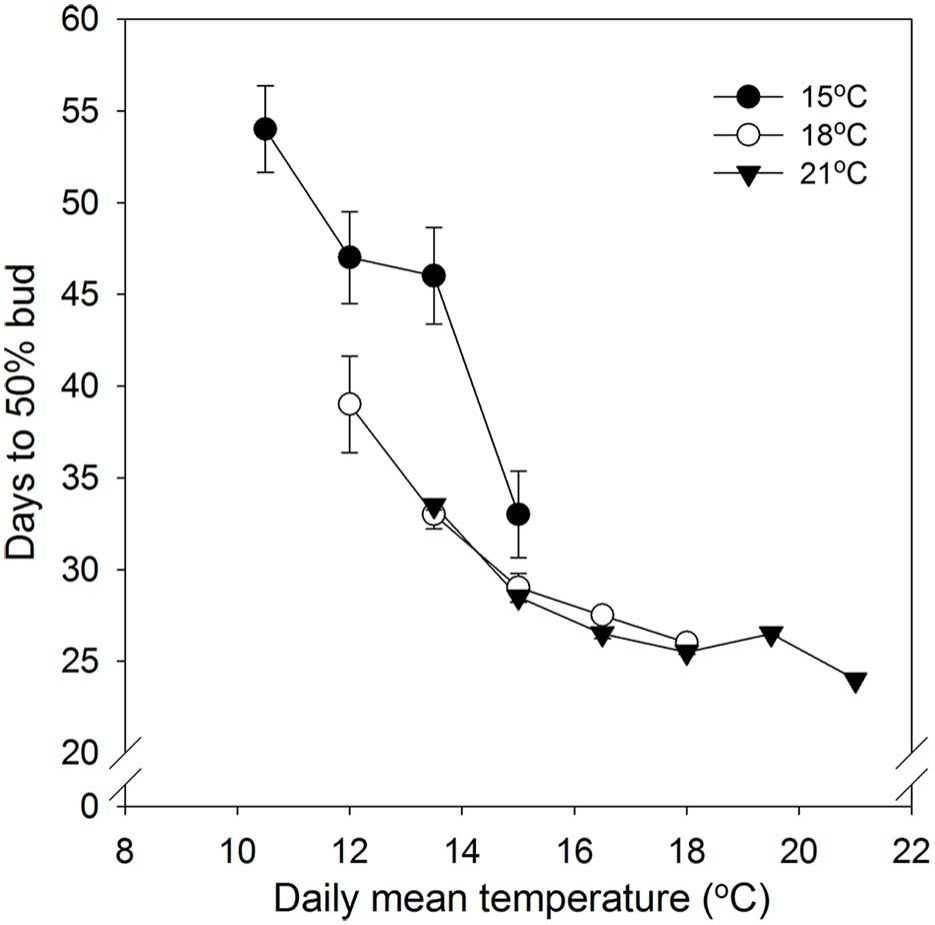
**Time to visible bud in 50% of seedlings of Norway spruce as affected by temperature during the short day period.** Results are means of all three provenances. Results are plotted against the daily mean temperature for the 3 day temperature treatments (15, 18, and 21°C, which were combined with NT of 6, 9, 12, 15, 18, and 21°C, i.e., up to 15, 18, and 21°C for the DT 15, 18, and 21°C, respectively). Vertical bars give the double SE (smaller than the symbol in treatments with day temperature of 21°C).

No specific effect of alternating DT and NT on time to 50% bud set was found. However, time course of bud set was enhanced with increasing temperature and correlated with the daily mean temperature (DMT; **Figure 1**). Under DTs of 18 and 21°C there was no significant difference between the same DMT regimes in time to visible bud in 50% of the seedlings (**Figure 1**). However, the DT of 15°C, combined with NT of 9, 12, and 15°C, delayed bud set significantly compared to the corresponding DMTs (12, 13.5, and 15°C) with higher DT, i.e., 18/6, 18/9, 18/12, 21/6, and 21/9°C DT/NT. Compared to higher DT, under DMT of 12°C and 13.5°C, a DT of 15°C delayed 50% bud set by 8 and 13 days, respectively. For the DMT of 15°C, the difference between the DT/NT 15/15 and 18/12°C was smaller, i.e., about 3 days.

Generally, time to 50% visible bud set was longer in seedlings of the intermediate provenance (M1, 64°N) than in the more northern (P1, 66°N) or more southern provenance (F1, 58°N; **Table 2**). The southernmost provenance (F1) usually showed 50% bud set earlier than the northernmost (P1), and this difference was most significant at low temperatures. Compared to the northernmost provenance, bud set was observed almost 7 days earlier for the southernmost at DT of 15°C, as averaged over all NT. For 18°C, the corresponding difference was small (although significant at *p* ≤ 0.05), i.e., only about 1 day, whereas for 21°C, there was no significant such difference.

**Table 2:** Days to 50% visible bud in seedlings of three provenances of Norway spruce as affected by temperature treatments applied during the short day period.

Provenance	15°C	18°C	21°C
P1 (66°N)	46.2^bc^	30.3^b^	26.9^a^
M1 (64°N)	48.9^b^	33.5^c^	28.4^b^
F1 (58°N)	39.4^a^	29.4^a^	26.5^a^

*Results are means of all night temperatures (NT) at the indicated day temperatures (NT 6, 9, 12, and 15°C for 15°C; 6, 9, 12, 15, and 18°C for 18°C; and 6, 9, 12, 15, 18, and 21°C for 21°C). In each column, values without a common letter are significantly different at *p* ≤ 0.05.*

All seedlings had ceased elongation growth within 4 weeks in SD. However, most of the plants grown at treatments with DT of 15°C and at treatments with low NT (6 and 9°C) combined with 18 or 21°C as DT, lacked a visible, terminal bud when transferred to forcing conditions (LD, 18°C, 24 h photoperiod) after 4 weeks in SD. In these plants, bud development continued under the LD conditions, and all the seedlings had visible buds 10–14 days after transfer to the forcing conditions. Initiation of new growth took place first after the development of visible bud was completed.

### EFFECT OF DAY–NIGHT TEMPERATURE COMBINATIONS DURING SD ON BUD BURST

**FIGURE 2:**
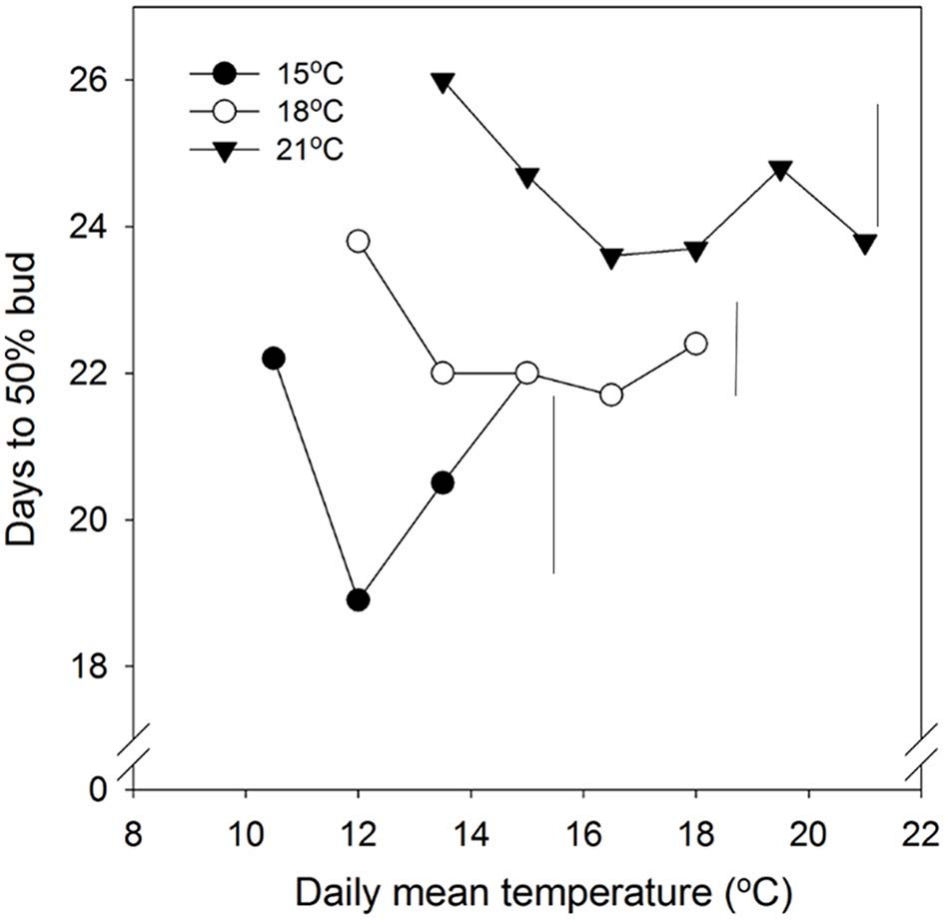
**Time to 50% bud burst in seedlings of Norway spruce as affected by temperature during short day treatment.** Results are means of all three provenances, and are plotted against the daily mean temperature for the 3 day temperature treatments (15, 18, and 21°C, which were combined with NT of 6, 9, 12, 15, 18, and 21°C, i.e., up to 15, 18, and 21°C for the DT 15, 18, and 21°C, respectively). Seedlings were not chilled before forcing (18°C, 24 h photoperiod). Vertical bars give the critical difference (*P* = 0.05) for night temperatures (NT) within the 3 day temperatures.

**FIGURE 3:**
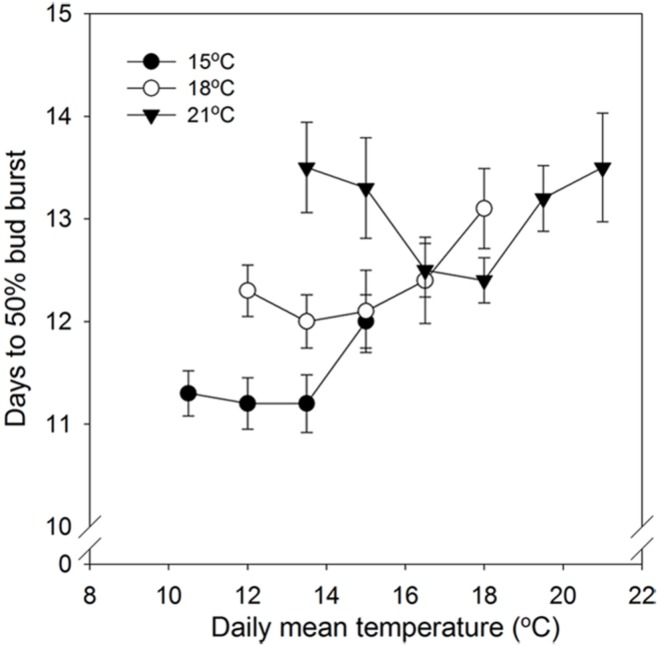
**Time to 50% bud burst in seedlings of Norway spruce as affected by temperature during short day treatment.** Results are means of all three provenances, and are plotted against the daily mean temperature for the 3 day temperature treatments (15, 18, and 21°C, which were combined with NT of 6, 9, 12, 15, 18, and 21°C, i.e., up to 15, 18, and 21°C for the DT 15, 18, and 21°C, respectively), all provided during 12 weeks of SD. Seedlings were chilled (means of 3, 6, and 9 weeks of chilling) before forcing (18°C, 24 h photoperiod). Vertical bars give the SE.

**FIGURE 4:**
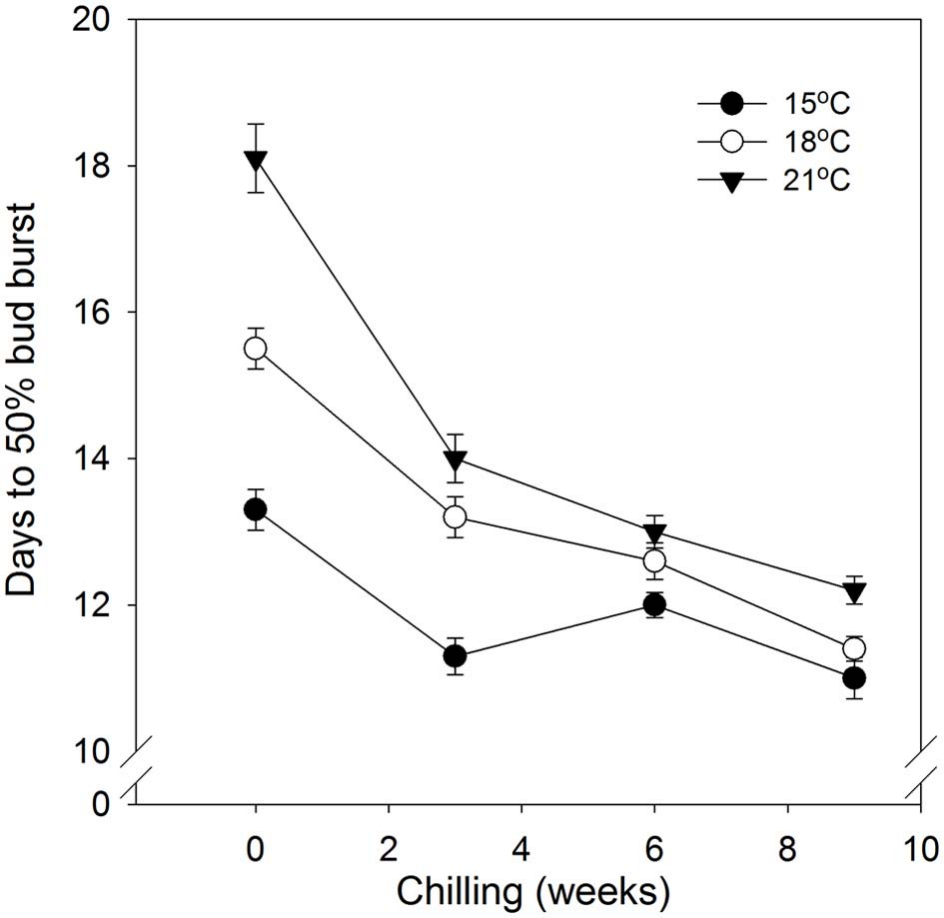
**Time to 50% bud burst in seedlings of Norway spruce as affected by chilling.** Results are means of all three provenances, and means of all NT at the indicated day temperatures (NT 6, 9, 12, and 15°C for 15°C; 6, 9, 12, 15, and 18°C for 18°C; and 6, 9, 12, 15, 18, and 21°C for 21°C), all provided during 12 weeks of SD. Vertical bars give the double SE.

In general, time to 50% bud burst increased with increasing temperature applied during the SD period (**Figure 2**; **Tables 3 and 4**). In un-chilled plants, exposure to a DT of 21°C under SD resulted in later bud burst, i.e., 24–26 days after transfer to forcing, compared to treatments with lower DT where bud burst was observed after 19–22 days, except 18/6°C DT/NT, which showed bud burst after 24 days (as averaged for the three provenances; **Figure 2**). In addition, there was a significant effect of NT. Bud burst was fastest in seedlings exposed to intermediate NT, irrespective of DT, and bud burst was delayed both by lower (6°C) and higher NT. In the un-chilled plants, the largest differences between NTs were observed for the DT of 15°C, for which the NT of 9°C resulted in the most rapid bud burst, (after about 19 days of forcing), compared to the slowest (after about 22 days) for NTs of 6°C and 15°C (**Figure 2**). The effect of temperature treatment applied during the SD period was significant even after chilling (**Figure 3**). The situation with slowest bud burst for higher and lower temperature under SD, compared to intermediate, was generally similar to in un-chilled plants (**Figure 3**). Also, like for un-chilled plants, the time to 50% bud burst in chilled plants increased with increasing temperature, but this relationship was most clear for the low NTs provided under SD. Generally, time to 50% bud burst decreased with increasing duration of chilling (**Figure 4**). Differences between provenances remained also after chilling; bud burst was earliest in the northernmost provenance P1 and latest in southernmost provenance F1 (**Table 4**).

Bud burst occurred in plants transferred directly to forcing conditions of LD and 18°C (no chilling). Time to 50% bud burst was then significantly reduced with increasing duration of the SD treatment (**Table 3**). After 8 and 12 weeks of SD, bud burst occurred about 21 and 15 days earlier than after 4 weeks of SD, as averaged for all three provenances at DT 18°C (all NT). The delay in bud burst after 4 weeks of SD was particularly prevalent under the lowest DT (15°C) and the lowest NTs combined with higher DT (data not shown), which might, as stated above, be at least partly due to that bud set was not complete after 4 weeks of SD. In un-chilled seedlings bud burst was latest in the southernmost provenance, and earliest in the northernmost provenance (**Table 3**). When exposed to 4 weeks of SD, bud burst then occurred about 14 and 10 days later in the southernmost provenance compared to the northernmost and intermediate, respectively. Differences between the provenances were reduced with an increasing duration of the SD treatment. After 8 weeks of SD, the southernmost provenance exhibited bud burst 3.2 and 2.5 days later than the northern and intermediate, respectively. After 12 weeks no significant differences between the provenances in un-chilled seedlings were observed.

**Table 3:** Days to 50% bud burst in seedlings of three provenances of Norway spruce as affected by duration of short day treatment (4, 8, or 12 weeks).

Provenance	4 weeks	8 weeks	12 weeks
P1 (66°N)	27.6^a^	16.9^a^	15.7^a^
M1 (64°N)	31.8^b^	17.6^a^	15.2^a^
F1 (58°N)	41.3^c^	20.1^b^	15.5^a^

*Results are means of temperature treatments 18/6, 18/9, 18/12, 18/15, and 18/18°C. Plants were transferred directly from short days to forcing conditions of 24 h photoperiod and 18°C (no chilling). In each column, values without a common letter are significantly different at *p* ≤ 0.05.*

In chilled seedlings latest bud burst was observed also in the southernmost provenance (**Table 4**). The difference between the southernmost and the two other provenances was generally significant (except the southern and intermediate at DT of 15°C), while the difference between the northernmost and the intermediate provenance was significant only in treatments with DT of 15°C but not 18°C and 21°C. For SD treatments of 21, 18, and 15°C (averaged over all NT), chilled seedlings of the southern provenance showed bud burst 2.1, 1.5, and 1.2 days later than the northern provenance, respectively.

**Table 4:** Days to 50% bud burst in seedlings of three provenances of Norway spruce as affected by temperature treatments applied during the short day period.

Provenance	15°C	18°C	21°C
P1 (66°N)	10.7^a^	11.7^a^	12.2^a^
M1 (64°N)	11.7^b^	12.2^a^	12.7^a^
F1 (58°N)	11.9^b^	13.2^b^	14.3^b^

*Results are means of all NT at the indicated day temperatures (NT 6, 9, 12, and 15°C for 15°C; 6, 9, 12, 15, and 18 for 18°C; and 6, 9, 12, 15, 18, and 21°C for 21°C). After short day treatment of 12 weeks, seedlings were chilled for 3, 6, or 9 weeks (means of all three treatments) before transfer to forcing conditions of 24 h photoperiod and 18°C. In each column, values without a common letter are significantly different at *p* ≤ 0.05.*

### BUD ANATOMY DEPENDS ON TEMPERATURE REGIME DURING SD EXPOSURE

**FIGURE 5:**
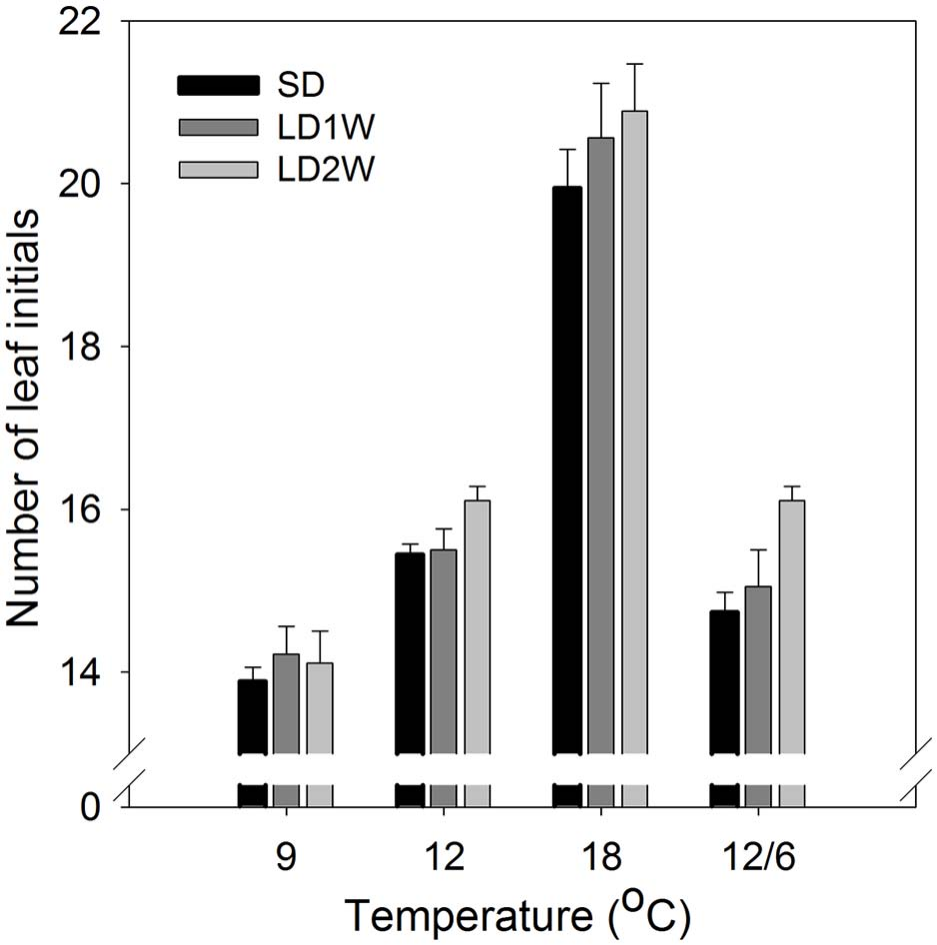
**Number of leaf initials in terminal buds of seedlings of Norway spruce after exposure to different temperature conditions (constant 9, 12, or 18°C or 12/6°C day/night temperature) under 8 weeks of short day conditions of 12 h photoperiod as well as one and 2 weeks after transfer to forcing conditions of long days and 18°C.** Results are mean of all three provenances, each with 3 plants per temperature treatment. Vertical bars show the SE.

**FIGURE 6:**
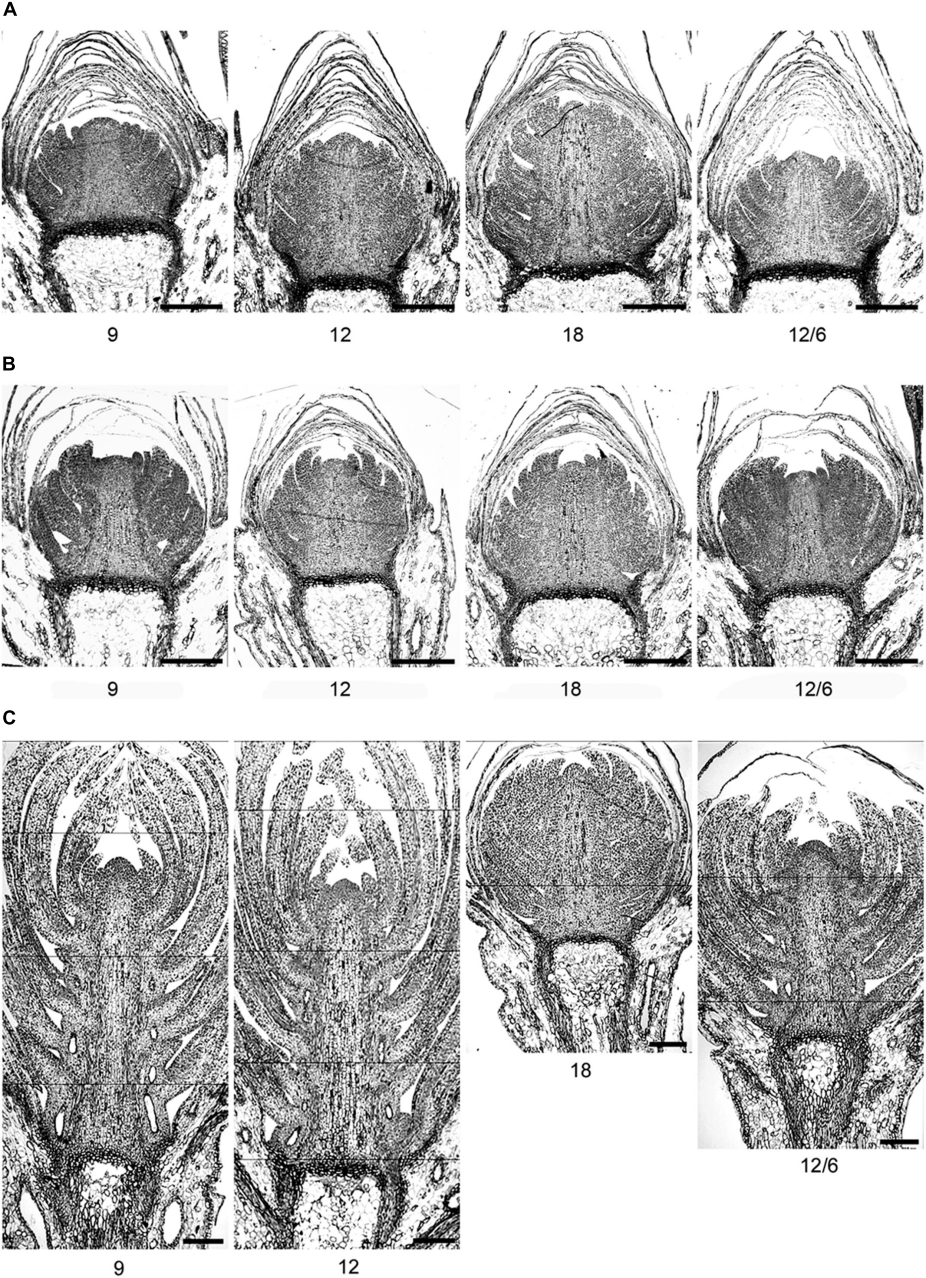
**Light micrographs of terminal buds in seedlings of Norway spruce after **(A)** exposure to different temperature conditions (constant 9, 12, or 18°C or 12/6°C day/night temperature) under 8 weeks of short day conditions of 12 h photoperiod and **(B)** one and **(C)** 2 weeks after re-transfer to 24 h photoperiod and 18°C after 8 weeks of short days**.

The number of leaf initials in SD-induced terminal buds (after 8 weeks of SD) was significantly affected by the temperature regime during the SD treatment (**Figures 5 and 6**; *p* ≤ 0.0001), but no significant difference between the three studied provenances was observed. In plants exposed to constant 18°C, the number of leaf initials (on average 19.1) was significantly higher compared to constant 9°C (13.3 leaf initials), 12°C (15.4 leaf initials), or 12/6°C DT/NT (14.1 leaf initials). There were no significant difference between constant 12°C and 12/6°C DT/NT, indicating no effect of NT at these DT/NT combinations. Also, there was no significant difference in number of leaf initials for the two treatments with the same DMT of 9°C, i.e., constant 9°C and 12/6°C DT/NT. Two weeks after re-transfer of the SD-exposed plants to forcing conditions of LD of a 24 h photoperiod and 18°C, the number of leaf initials had increased significantly by on average 1.3 leaf initials compared to at the end of the SD period of 8 weeks (**Figure 5**; *p* ≤ 0.0001). The most pronounced increase in number of leaf initials (2 leaf initials) was observed in the plants that were exposed to 12/6°C DT/NT compared to the plants exposed to the same DT (constant 12°C) or same daily DMT (9°C). Like after the SD period of 8 weeks, there was no significant difference in number of leaf initials between the studied provenances one and 2 weeks after the transfer to forcing conditions.

## DISCUSSION

Although temperature under SD exposure of woody species has been shown to modulate the timing of bud set and subsequent bud break, most studies have tested constant temperatures or a limited number of DT-NT combinations only, and few studies have systematically addressed effects of diurnal temperature fluctuation. To shed light on effects of diurnal temperature variation on bud development and depth of dormancy in Norway spruce, we have investigated the effect of a wide range of different DT-NT combinations on bud set, subsequent bud burst as well as bud anatomy. We demonstrate here that the effect of DT is significantly modified by NT in a complex manner, and that the temperature regime during SD significantly affects the bud development.

### EFFECT OF DIFFERENT DAY–NIGHT TEMPERATURE COMBINATIONS DURING SD ON BUD SET

No specific effect of alternating DT and NT on time to 50% bud set under SD was observed. However, but set correlated with the DMT with accelerated bud set with increasing DMT (**Figure 1**; **Table 2**). This is consistent with previously observed acceleration of bud set with increasing temperature in a number of tree species, which in most cases were grown under constant temperatures or in some cases a limited number of DT-NT combinations only (Heide, 2003; Junttila et al., 2003; Søgaard et al., 2008; Kalcsits et al., 2009a; Tanino et al., 2010 and references therein. However, although time to visible bud in 50% of the seedlings in our study generally correlated with the DMT under SD, days to bud set also depended on the specific DT (**Figure 1**). Whereas for DT of 18 and 21°C there were no significant differences in time to visible bud between equal DMTs, a DT of 15°C delayed bud set substantially compared to higher DT for the DMTs 12 and 13.5°C (NT 9°C and 12°C at 15°C DT). For the DMT 15°C, there was also some delay, but smaller, for DT 15°C compared to higher DTs. Thus, the combination of the DT of 15°C with a NT 15°C, accelerated bud set compared to lower NTs at the same DT.

By employing a substantial number of DT-NT combinations resulting in eight different DMTs, our results clearly demonstrate that bud set is a temperature-dependent process, and that, although the DMT is an important determinant of the time to bud formation, the actual temperature combination is also important. Low DT delays the process, but there is also an interaction with NT in that low NT also delays bud set. Acceleration of bud set by higher NT is similar to the situation reported for poplar hybrids, for which four different DT-NT combinations were tested (Tanino et al., 2010). Delay in bud set by low NT as well as low DT may be a consequence of reduced rates of metabolism in the lower temperatures. In Norway spruce energy metabolism was shown to be stimulated initially after transfer of seedlings to SD at 18°C, before decreasing under long-term SD exposure (Lee et al., 2014). Since low temperature (including low NT) is well known to decrease respiration (Saxe et al., 2001), delay in bud formation under low NT might well be at least partly attributed to reduced respiration and thus shortage in energy and metabolites required for bud formation. Furthermore, low temperature in presence of light induces photoinhibition of photosynthesis and ultimately photooxidative stress (Öquist and Huner, 2003). Also, low NT was shown to increase photoinhibition in *Abies lasiocarpa* seedlings (Germino and Smith, 1999). Thus, in the present study low DT (15°C) and possibly also low NT might enhance photoinhibition, which might then reduce the capacity for the comprehensive modulation of the metabolome associated with bud set in seedlings of Norway spruce (Lee et al., 2014). However, different species and even ecotypes may differ in their sensitivity to photoinhibition. Differences in effect of temperature on bud set in ecotypes of dogwood (*Cornus sericea*) was recently demonstrated to be associated with different tolerance to photoinhibition (Tanino et al., 2010, 2014). Furthermore, decreased water mobility during SD-induced dormancy and temperature modulation of the process, imply that changes in water mobility are closely associated with dormancy state (Kalcsits et al., 2009b). Thus, differences in time to bud set between temperature regimes in this study might well be associated with differences in water mobility. Furthermore, daylength perception/action associated with the phytochrome system in woody species is apparently modulated by temperature (Olsen et al., 1997a; Mølmann et al., 2005). The effect of temperature on SD-induced bud set observed here might thus be attributed to effect of temperature on phytochrome action. In this respect temperature may, e.g., affect dark reversion of Pfr with warm temperature accelerating reversion (references in Tanino et al., 2010) and/or act on downstream signaling components such as PHYTOCHROME INTERACTING FACTOR 4 (PIF4), which, at least in *Arabidopsis thaliana*, show increased gene expression with elevated temperature (Koini et al., 2009; Stavang et al., 2009). It has also been shown that morphogenetic temperature responses differ during day and night, including in woody species (Went, 1944; references in Tanino et al., 2010 and Olsen and Lee, 2011). Since SD-induced growth cessation and bud set is associated with down-regulation of the growth-promoting hormones gibberellin (GA) and auxin (Moritz, 1995; Olsen et al., 1995a,b, 1997a,b), it might be hypothesized that temperature also play a role in regulation of this hormones, like shown in herbaceous plants (Stavang et al., 2005, 2009). However, increased levels of these hormones upon a temperature increase, as shown in herbaceous plants, and reduced levels under SD-induced bud formation in woody species are not easily integrated in a model for acceleration of bud set with increasing temperature. Furthermore, in woody species, including Norway spruce seedlings, the growth inhibiting hormone abscisic acid (ABA) is up-regulated in SD (Lee et al., 2014). Since ABA levels are also well known to be affected by temperature (Welling and Palva, 2006), it might well be that delayed bud set in response to low temperature is associated with higher levels of ABA. Although DT and NT have been shown to regulate GA and auxin metabolism differentially in herbaceous plants with respect to shoot elongation (Thingnæs et al., 2003; Stavang et al., 2005), thermoperiodic regulation of hormone metabolism remains to be investigated in woody species.

Whereas growth cessation and bud set previously was shown to occur earlier in northern compared to southern provenances, at least when grown in growth chambers under constant temperature of 18°C and controlled light conditions (e.g., Mølmann et al., 2006), in the current study 50% bud set was generally observed earlier in the southernmost (F1) than the northernmost provenance (P1) when grown in DT 15°C or 18°C, as averaged over all NTs (**Table 2**). This was particularly pronounced in low temperatures. However, as compared to the more southern provenances, particularly at the lower temperatures the northernmost provenance commonly exhibited more variation between plants in the time to bud set with a few plants commonly showing the earliest bud set of all provenances. Although the reason for this remains elusive, it might be that bud set in some genotypes within the northernmost provenance is more sensitive to low temperature stress than others. Genotypic variations in temperature sensitivity of growth cessation and dormancy development has been reported also in hybrid poplar (Kalcsits et al., 2009a). Growth cessation or/and bud set in response to low temperatures, even under LD, has been observed in northern provenances of a range of woody species (Håbjørg, 1972a,b; Heide, 1974; Junttila, 1980; Mølmann et al., 2005; Svendsen et al., 2007). Nevertheless, for all provenances in this study, the acceleration of bud set with increasing DT and correlation with DMT were evident.

In spite of that all seedlings apparently had stopped elongation growth within 4 weeks in SD, the lack of buds at this time point for most plants grown under DT of 15°C irrespective of NT, and low NT (6 and 9°C) combined with DT of 18 and 21°C, underlines that bud development is strongly affected by temperature and is considerably delayed under low temperature conditions. Furthermore, the continuation of bud development in the plants without visible bud when transferred to forcing conditions, indicates that the developmental program resulting in formation of a terminal bud is not reversed when first initiated, or when first having passed a certain stage. Further development of leaf initials within the terminal bud of plants exposed to 12/6°C DT/NT under SD before transfer to forcing is consistent with this (See also section on discussion of microscopy data below).

### EFFECT OF DIFFERENT DAY–NIGHT TEMPERATURE COMBINATIONS DURING SD ON BUD BURST

As demonstrated also previously for several deciduous and coniferous species, including Norway spruce when grown under constant temperatures during SD treatment (Heide, 2003; Junttila et al., 2003; Søgaard et al., 2008; Kalcsits et al., 2009a; Tanino et al., 2010 and references therein), bud burst was delayed by increasing temperature during the SD-induced bud formation. Specifically, increasing DT generally delayed the bud flush in the present study (**Table 4**; **Figure 2**). The effect persisted even after chilling treatment (**Figure 3**). By employing a wide range of DT-NT combinations, the present results also demonstrate a significant effect of NT on time to bud burst, with generally fastest bud flush at intermediate temperatures, both in un-chilled and chilled plants (**Figures 2 and 3**). Thus, our results show that the effects of DTs are significantly modified by NT, although in a complex way. In contrast to under the intermediate temperatures, in lower (6°C) and higher NT bud set was delayed. It might well be that the delay in bud set at the NT of 6°C was due to the delayed bud development and continuation of bud development to a certain stage after the transfer to the forcing conditions, before the processes leading to bud burst could be initiated. Accordingly, in Norway spruce seedlings, effect of temperature on time course of bud burst involves both an effect on bud development itself (ontogenesis) and an effect on dormancy-related processes. The delay in bud flush at high temperatures compared to the intermediate temperatures might be due to that these intermediate (lower) temperatures could also act as chilling treatment hastening bud flush. It is well known that quiescent buds in woody species will flush only after exposure to a cumulative heat sum (Heide, 1993; Junttila and Hänninen, 2012). However, apparently there is considerable overlap between the temperatures that are effective in chilling and those that contribute to the heat sum (Cooke et al., 2012; Luedeling et al., 2013). Our results are in accordance with such a situation. Longer time to bud break without chilling, although young seedlings of Norway spruce does not have a strict chilling requirement, is consistent with that a greater heat sum is usually needed to re-initiate growth if the chilling period is sub-optimal.

Winter dormancy in seedlings of Norway spruce has previously been shown to be quite shallow in the sense that chilling is not required for dormancy alleviation, although hastening it (Nienstaedt, 1967; Worrall and Mergen, 1967; Søgaard et al., 2008). This was confirmed here as bud flushing occurred also in the plants transferred to the LD forcing conditions without any chilling treatment, although later than in the chilled plants (**Tables 3 and 4**). The fact that time to bud burst was significantly reduced with increasing duration of the SD treatment (**Table 3**) even without subsequent chilling, indicates that under extended SD the bud undergoes a developmental program, which decreases the depth of dormancy. The strongly delayed bud burst after 4 weeks of SD, compared to the more extended SD periods (8 and 12 weeks), was probably due to the fact that most plants grown at low DMT did not yet have a visible terminal bud after 4 weeks of SD but continued bud development after transfer to forcing conditions. The time needed for completion of bud formation thus also contributed to delayed initiation of new growth in these cases. It thus appears that bud development must reach a certain stage before the processes leading to bud burst/re-initiation of growth can be initiated, as suggested also by a study of buds of more mature (13 years old) field-grown trees of Norway spruce (Viherä-Aarnio et al., 2014).

Our results from chilled and un-chilled plants show similar differences between provenances with decreasing time to bud burst with increasing northern latitude of origin (**Tables 3 and 4**). Young plants of Norway spruce has been shown to exhibit a clinal variation in which southern and lowland provenances show later bud burst than more northern and high altitude provenances (Worrall and Mergen, 1967; Beuker, 1994; Søgaard et al., 2008). However, differences between provenances were reduced with increasing duration of the SD treatment (**Table 3**), but were larger after dormancy induction at high compared to lower DT (**Table 4**). Furthermore, as demonstrated by Søgaard et al. (2008), differences between provenances was larger in un-chilled compared to chilled plants (**Tables 3 and 4**).

### BUD ANATOMY DEPENDS ON TEMPERATURE REGIME DURING SD EXPOSURE

After initiation of bud scales, the organogenetic activity may continue for several weeks in SD (Owens and Molder, 1976; MacDonald and Owens, 1993). In accordance with the recordings of generally faster formation of visible buds at DT 18°C compared to a lower DT (**Table 2**), higher number of leaf initials (primordia) at 18°C than the lower temperatures (9 and 12°C; **Figures 5 and 6**) clearly demonstrates that bud set and rate of primordia production is a strongly temperature-dependent process. Furthermore, the delay in bud burst after higher compared to lower temperature during SD, correlates with the more well-developed buds (more primordia) under higher compared to lower temperatures under SD. The results from the microscopy analyses also confirm that bud development under SD correlates with the DMT, i.e., there was no significant difference in number of leaf initials for the two treatments with the same DMT of 9°C, i.e., constant 9°C and 12/6°C DT/NT. Although the studies of visible bud set indicated interaction between DT and NT, in that low NT delays bud set more under lower (15°C) compared to higher DT (18 and 21°C), there was no significant difference in number of leaf initials between constant 12°C and 12/6°C DT/NT, indicating no effect of NT at these DT/NT combinations. Continued formation of leaf initials after transfer to forcing conditions of LD and 18°C, may support the idea that bud formation might have to pass a certain stage before new elongation growth is initiated.

## CONCLUSION

Clearly, bud development is a temperature-dependent process and is strongly delayed under low temperature conditions. The present results demonstrate that the effect of DT on time to bud set is significantly modified by NT in a complex manner, although alternating DT and NT in itself does not affect time to bud set. Since time to visible bud in 50% of the seedlings correlated with the DMT under SD at DTs of 18 and 21°C, but not a DT of 15°C days to bud set apparently also depend on the specific DT, implying also that the effect of NT depends on the actual DT. Furthermore, whereas higher temperature under SD results in longer time to bud break after transfer to forcing conditions, our results also demonstrate that the effect of DT under SD is significantly modified by NT in a complex way, with fastest bud flush at intermediate temperatures. This might be due to a chilling effect of intermediate temperatures, which hasten bud break compared to higher temperatures, and delayed bud development to a stage where bud burst can occur, under lower temperatures. Thus, the time course of bud burst in Norway spruce seedlings appears to involve an effect on bud ontogenesis as well as an effect of dormancy-related processes.

The results also suggest that the duration of the SD treatment on bud burst is at least partly related to the development of the bud itself; Bud development must apparently reach a certain stage before the processes leading to bud burst are initiated. The results also indicate that low temperature during bud development had a larger effect on the southern than the northern provenance. In addition, our results confirm that seedlings of Norway spruce are able to flush in LD even without any chilling treatment. Furthermore, bud burst is delayed by high temperatures applied during SD exposure and this effect persists, although reduced, even after prolonged chilling.

## Conflict of Interest Statement

The authors declare that the research was conducted in the absence of any commercial or financial relationships that could be construed as a potential conflict of interest.
